# Rituximab as therapy to induce remission after relapse in ANCA-associated vasculitis

**DOI:** 10.1136/annrheumdis-2019-216863

**Published:** 2020-06-24

**Authors:** Rona M Smith, Rachel Bronwen Jones, Ulrich Specks, Simon Bond, Marianna Nodale, Reem Aljayyousi, Jacqueline Andrews, Annette Bruchfeld, Brian Camilleri, Simon Carette, Chee Kay Cheung, Vimal Derebail, Tim Doulton, Lindsy Forbess, Shouichi Fujimoto, Shunsuke Furuta, Ora Gewurz-Singer, Lorraine Harper, Toshiko Ito-Ihara, Nader Khalidi, Rainer Klocke, Curry Koening, Yoshinori Komagata, Carol Langford, Peter Lanyon, Raashid Ahmed Luqmani, Hirofumi Makino, Carole McAlear, Paul Monach, Larry W Moreland, Kim Mynard, Patrick Nachman, Christian Pagnoux, Fiona Pearce, Chen Au Peh, Charles Pusey, Dwarakanathan Ranganathan, Rennie L Rhee, Robert Spiera, Antoine G Sreih, Vladimir Tesar, Giles Walters, Michael H Weisman, Caroline Wroe, Peter Merkel, David Jayne, RITAZAREM co-investigators, Y Arimura

**Affiliations:** 1 University of Cambridge, Cambridge, UK; 2 Cambridge University Hospitals NHS Foundation Trust, Cambridge, UK; 3 Mayo Clinic, Rochester, Minnesota, USA; 4 Cambridge Clinical Trials Unit, Cambridge University Hospitals NHS Foundation Trust, Cambridge, UK; 5 University Hospitals of Leicester NHS Trust, Leicester, Leicester, UK; 6 NIHR Leeds Musculoskeletal Biomedical Research Unit, Leeds Teaching Hospitals Trust, Leeds, UK; 7 Department of Renal Medicine, Karolinska University Hospital and Karolinska Institute, Stockholm, Sweden; 8 Ipswich Hospital NHS Trust, Ipswich, UK; 9 University of Toronto, Toronto, Ontario, Canada; 10 University of Leicester, Leicester, UK; 11 University of North Carolina, Chapel Hill, North Carolina, USA; 12 East Kent Hospitals University NHS Foundation Trust, Canterbury, Kent, UK; 13 Cedars-Sinai Medical Center, Los Angeles, California, USA; 14 University of Miyazaki, Miyazaki, Japan; 15 Chiba University, Chiba, Japan; 16 University of Michigan, Ann Arbor, Michigan, USA; 17 University of Birmingham, Birmingham, UK; 18 Kyoto University, Kyoto, Japan; 19 McMaster University, Hamilton, Ontario, Canada; 20 Dudley Group NHS Foundation Trust, Dudley, West Midlands, UK; 21 University of Utah Vasculitis Center, Salt Lake City, Utah, USA; 22 Kyorin University School of Medicine, Tokyo, Japan; 23 Cleveland Clinic Foundation, Cleveland, Ohio, USA; 24 Rheumatology, Nottingham University Hospital, Nottingham, UK; 25 Nuffield Department of Orthopaedics, Rheumatology and Musculoskeletal Science (NDORMs), University of Oxford, Oxford, UK; 26 Okayama Universty Hospital, Okayama, Japan; 27 Division of Rheumatology, University of Pennsylvania Perelman School of Medicine, Philadelphia, Pennsylvania, USA; 28 Division of Rheumatology, VA Boston Healthcare System, West Roxbury, Massachusetts, USA; 29 University of Pittsburg, Pittsburg, Pennsylvania, USA; 30 Mount Sinai Hospital, University of Toronto, Toronto, Ontario, Canada; 31 Nottingham University Hospitals NHS Trust, Nottingham, Nottingham, UK; 32 Royal Adelaide Hospital, Adelaide, South Australia, Australia; 33 Imperial College London, London, UK; 34 Royal Brisbane and Women's Hospital, Herston, Queensland, Australia; 35 Perelman School of Medicine at the University of Pennsylvania, Philadelphia, Pennsylvania, USA; 36 HSS, New York, New York, USA; 37 University of Pennsylvania Perelman School of Medicine, Philadelphia, Pennsylvania, USA; 38 Department of Nephrology, Charles University, Prague, Czech Republic; 39 Canberra Hospital, Canberra, Australian Capital Territory, Australia; 40 South Tees Hospitals NHS Foundation Trust, Middlesbrough, Middlesbrough, UK; 41 University of Pennsylvania, Philadelphia, Pennsylvania, USA

**Keywords:** B cells, granulomatosis with polyangiitis, treatment, systemic vasculitis

## Abstract

**Objectives:**

Evaluation of rituximab and glucocorticoids as therapy to induce remission after relapse in ANCA-associated vasculitis (AAV) in a prospective observational cohort of patients enrolled into the induction phase of the RITAZAREM trial.

**Methods:**

Patients relapsing with granulomatosis with polyangiitis or microscopic polyangiitis were prospectively enrolled and received remission-induction therapy with rituximab (4×375 mg/m^2^) and a higher or lower dose glucocorticoid regimen, depending on physician choice: reducing from either 1 mg/kg/day or 0.5 mg/kg/day to 10 mg/day by 4 months. Patients in this cohort achieving remission were subsequently randomised to receive one of two regimens to prevent relapse.

**Results:**

188 patients were studied: 95/188 (51%) men, median age 59 years (range 19–89), prior disease duration 5.0 years (range 0.4–34.5). 149/188 (79%) had previously received cyclophosphamide and 67/188 (36%) rituximab. 119/188 (63%) of relapses had at least one major disease activity item, and 54/188 (29%) received the higher dose glucocorticoid regimen. 171/188 (90%) patients achieved remission by 4 months. Only six patients (3.2% of the study population) did not achieve disease control at month 4. Four patients died in the induction phase due to pneumonia (2), cerebrovascular accident (1), and active vasculitis (1). 41 severe adverse events occurred in 27 patients, including 13 severe infections.

**Conclusions:**

This large prospective cohort of patients with relapsing AAV treated with rituximab in conjunction with glucocorticoids demonstrated a high level of efficacy for the reinduction of remission in patients with AAV who have relapsed, with a similar safety profile to previous studies.

**Key messages**

**What is already known about this subject?**

- Rituximab is increasingly being used as a remission induction agent in ANCA-associated vasculitis.

**What does this study add?**

- This large prospective cohort provides further efficacy and safety data for the use of rituximab in patients specifically with relapsing disease.

**How might this impact on clinical practice or future developments?**

- Rituximab in conjunction with glucocorticoids is now an established induction strategy in ANCA-associated vasculitis.

## Introduction

Granulomatosis with polyangiitis (GPA) and microscopic polyangiitis (MPA) are the major subgroups of antineutrophil cytoplasmic antibody (ANCA)-associated vasculitis (AAV). These conditions are characterised by leucocyte infiltration of blood vessel walls, fibrinoid necrosis and vascular damage and are usually associated with the presence of circulating ANCA.^1^

Prior to the availability of effective treatment, AAV had a mortality of 93% within 2 years, primarily due to renal and respiratory failure.^2^ The introduction of glucocorticoids and cyclophosphamide, which became established treatment for this disease in the 1980s, markedly improved survival, inducing remission at 1 year in approximately 80% of patients. However, relapsing disease is common with over 50% of patients experiencing a relapse within 5 years and the majority suffering treatment-related toxicity.^3–5^

B-lymphocytes have been implicated in the pathogenesis of AAV. Rituximab is a murine/human chimeric monoclonal antibody directed against the CD20 antigen found on the surface of B-lymphocytes and results in B cell depletion. Rituximab was shown to be non-inferior to cyclophosphamide for induction of remission in AAV and superior to cyclophosphamide for the treatment of relapsing disease.^6 7^ Rituximab became a licenced therapy for remission induction of AAV in 2011.

Fixed-interval, repeat-dosing of rituximab was shown to be superior to azathioprine as a maintenance strategy following induction of remission cyclophosphamide in a trial of 117 patients with predominantly newly diagnosed AAV.^8^ The optimal strategy to maintain remission following induction of remission with rituximab, especially for treatment of relapse, is not clear. RITAZAREM was an international, randomised, controlled trial designed to assess whether rituximab is superior to azathioprine for the maintenance of remission following induction of remission with rituximab and glucocorticoids in patients with relapsing AAV. In this trial, fixed-interval, repeat doses of rituximab were compared with daily azathioprine for maintenance of remission.

Since all patients received rituximab for induction of remission in the RITAZAREM trial, this is the largest reported prospective cohort of patients with relapsing AAV to receive treatment with rituximab for induction of remission. This first report outlines the efficacy and safety of rituximab with either higher or lower dose glucocorticoids for induction of remission in a large prospective cohort of patients with relapsing AAV.

## Methods

The details of the RITAZAREM protocol have been previously published.^9^ In summary, RITAZAREM trial has three phases:

1. An *induction phase* (months 0–4): eligible patients enrolled at time of disease relapse received rituximab (4 weekly doses of 375 mg/m^2^) and glucocorticoids.
2. A *maintenance phase* (months 4–24): 4 months after enrolment, participants who achieved remission (defined as a Birmingham Vasculitis Activity Score for Wegener’s granulomatosis (BVAS/WG) ≤1 and prednisone/prednisolone dose ≤10 mg/day) were randomised in 1:1 ratio to receive 1000 mg rituximab at 4 monthly fixed intervals or daily azathioprine (2 mg/kg/day).
3. A *follow-up phase:* clinical follow-up after completion of therapy with either rituximab or azathioprine (minimum of 12, maximum of 24 months).

This paper reports on the first, induction phase of the trial, prior to randomisation.

### Participants

Participants were aged over 15 years and had a diagnosis of GPA or MPA according to Chapel Hill Consensus Conference definitions^10^ and a current or historical positive test for PR3-ANCA or MPO-ANCA. All patients had disease relapse defined by one major or three minor disease activity items on the BVAS/WG and had previously achieved remission following at least 3 months of induction therapy, with a combination of glucocorticoids and an immunosuppressive agent (cyclophosphamide, rituximab, methotrexate or mycophenolate mofetil).

Key exclusion criteria included the receipt of any biological B cell depleting agents within the previous 6 months, alemtuzumab or antithymocyte globulin within the previous 12 months, or intravenously administered immunoglobulin, plasma exchange or anti-tumour necrosis factor (TNF) treatment within the previous 3 months. Patients with other multisystem autoimmune diseases, such as eosinophilic granulomatous with polyangiitis, systemic lupus erythematosus, antiglomerular basement membrane disease or cryoglobulinaemic vasculitis or history of malignancy within the past 5 years were also excluded.

Participants were recruited from 29 centres in seven countries.

### Interventions and induction therapy

#### Rituximab

Rituximab 375 mg/m^2^/week was administered in four doses.

#### Glucocorticoids

Investigators chose from one of two glucocorticoid regimens taking into consideration disease severity and local prescribing practices. Schedule 1A had a glucocorticoid starting dose of 1 mg/kg/day (maximum 60 mg daily) and 1B had a starting dose of 0.5 mg/kg/day (maximum 30 mg daily), both tapering to 10 mg daily by month 4. Deviation from the protocol-specified tapering glucocorticoid regimen was defined as a 25% higher or lower glucocorticoid dose, averaged over 2 weeks. Patients could also receive a maximum cumulative dose of 3000 mg intravenous methylprednisolone, between 14 days prior to enrolment and 7 days after enrolment.

#### Other treatments

Prophylaxis to prevent *pneumocystis (carinii) jiroveci* infection and/or to prevent osteoporosis were recommended according to local practice. Plasma exchange could be administered during the induction period following local practice. However, rituximab was not administered within 48 hours before a plasma exchange treatment.

#### Assessments

Evaluations (including clinical, biochemical and patient-reported outcomes) were performed at 0, 1.5, 3 and 4 months.

#### Power calculation

Enrolment was set to be open until at least 160 patients were randomised at their month 4 visits. It was anticipated that 190 patients would be required in order to randomise 160 patients. Details of how the sample size was determined have been previously published.^9^

#### Definitions

Remission was defined as a BVAS/WG of 1 or less with a prednisone/prednisolone dose of 10 mg/day or less by 4 months.

#### Statistical methods

Continuous variables are expressed as medians and IQRs. Categorical variables are presented as percentages and frequencies. A set of univariate logistic regression analyses to predict remission at month 4 for candidate factors was performed. Estimates of marginal ORs, with 95% CIs and p values are presented. The statistical comparisons were not formally powered or prespecified in the protocol so these results must be interpreted with caution. Data were analysed using R V.3.6.1.

## Results

### Baseline demographics

**Figure 1:**
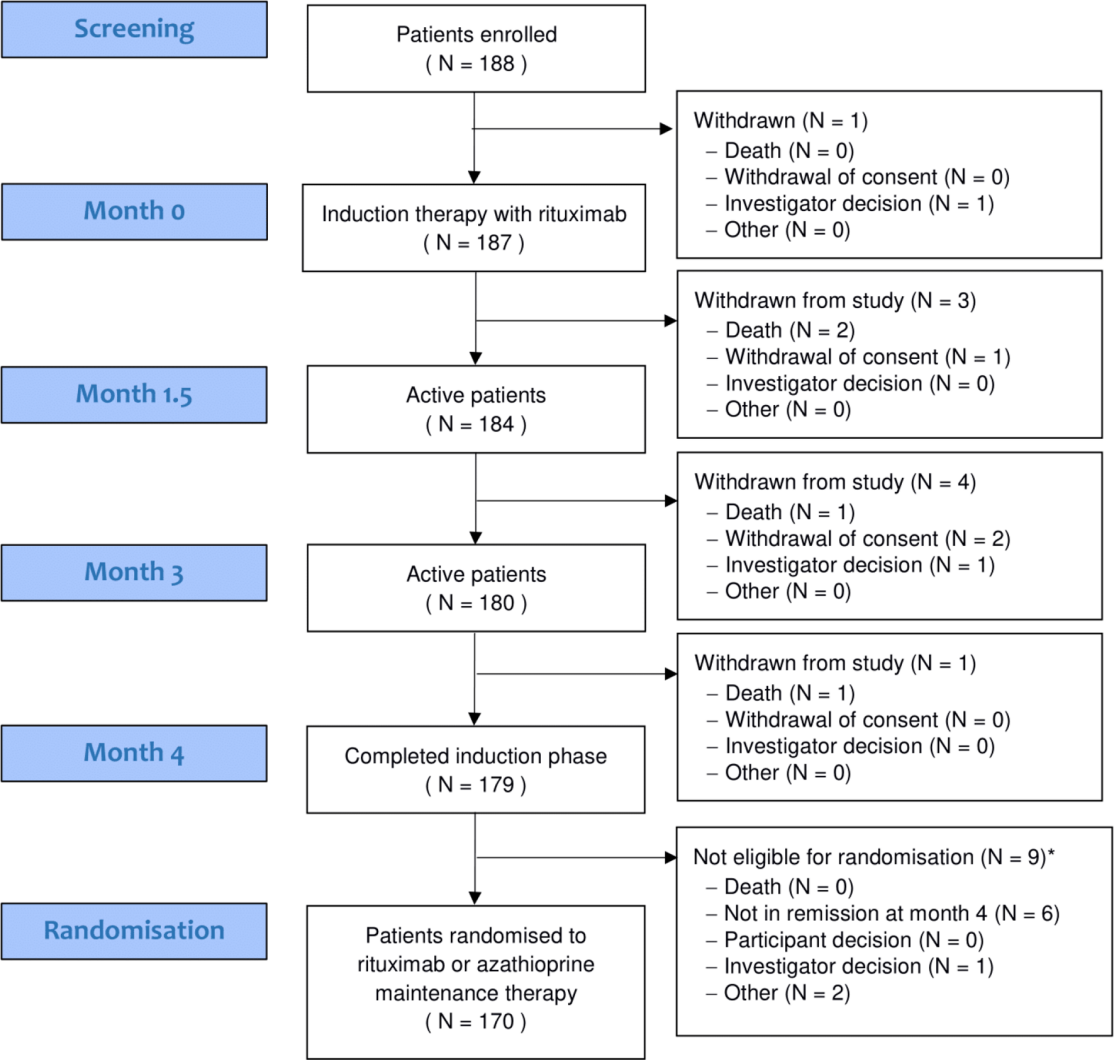
Consort diagram.

188 patients were enrolled into the trial. Patient disposition throughout the 4-month induction period is shown in the consort diagram (figure 1) and baseline demographics in table 1. Ninety-five out of 188 (51%) patients were male, with a median age of 59 years (range 19–89) and prior disease duration of 5.0 years (range 0.4–34.5). One hundred and forty-nine (79%) patients had previously received cyclophosphamide (median dose 9 g (range 0.15–301) and 67/188 (36%) had received rituximab (median dose 3910 mg (range 1000–16000)). At enrolment, 60/188 (32%) patients were on an oral immunosuppressive agent: (35/188 (19%) azathioprine; 12/188 (6%) mycophenolate mofetil; and 13/188 (7%) methotrexate), each of which were stopped as per protocol. One hundred and thirty-seven out of 188 (73%) had a history of a positive test for PR3-ANCA and 51/188 (37%) for MPO-ANCA. One hundred and nineteen out of 188 (63%) of relapses had at least one major disease activity item, and 54/188 (29%) patients received the higher dose glucocorticoid regimen. The median BVAS/WG at enrolment was 5 (range 3–14). Distribution of baseline disease manifestations included: ear, nose and throat: 120/187 (64.2%) patients, renal: 88 (47.1%) patients and respiratory involvement: 69 (36.9%) patients.

**Table 1:** Baseline demographics

	Total (n=188)
Age, years: median (range)	59 (19–89)
Male, number (%)	95 (51)
Race, number (%)	
White	168 (89.4)
Asian	13 (6.9)
Hispanic	3 (1.6)
Black	1 (0.5)
Other	3 (1.6)
Disease duration, years: median (range)	5.0 (0.4–34.5)
Prior treatment with cyclophosphamide	
Number of patients (%)	149 (79.3)
Cumulative dose, grams (g): median (range)	9 (0.15–301)
Prior rituximab therapy	
Number of patients (%)	67 (35.6)
Cumulative dose, grams (g): median (range)	3910 (1000–16000)
Glucocorticoid induction regimen, number (%)	
1 mg/kg/day starting dose (1A)	54 (28.7)
0.5 mg/kg/day starting dose (1B)	134 (71.3)
ANCA type, number (%)	
Antiproteinase 3	137 (72.9)
Antimyeloperoxidase	51 (27.1)
Relapse type on entry into trial, number (%)	
Severe	119 (63.3)
Non-severe	69 (36.7)
BVAS/WG: median (range)	5 (3–14)

ANCA, antineutrophil cytoplasmic antibody; BVAS/WG, Birmingham Vasculitis Activity Score for Wegener's granulomatosis.

The median number of body systems previously affected by vasculitis was 5 (range 0–10). Prior organ involvement included renal in 127/188 (67.6%) patients, lung in 115/188 (61.2%) patients and ear nose and throat in 138/188 (73.4%) patients. Hypertension was common, affecting 93/199 (49.5%) patients. Twenty-three out of 188 (12.2%) patients had diabetes mellitus at enrolment; 29/188 (15.4%) had chronic lung disease and 20/188 (10.6%) had previously suffered from malignancy.

### Treatment exposure

The median total dose of rituximab in the induction phase was 2937 mg (range 1552–4320 mg) and cumulative oral glucocorticoid exposure in the 4-month induction phase was 3010 mg (2485–7875 mg) in the 1A higher dose induction regimen and 1960 mg (1715–3535 mg) in the 1B lower dose induction regimen. There was no difference in cumulative glucocorticoid exposure between patients that achieved and did not achieve remission (median dose 1960 mg in both groups (1A range: 1715–3010; 1B range: 1715–7875). Twenty-five per cent of patients deviated from the specified glucocorticoid tapering regimen at some point in the induction phase.

### Disease response

**Figure 2:**
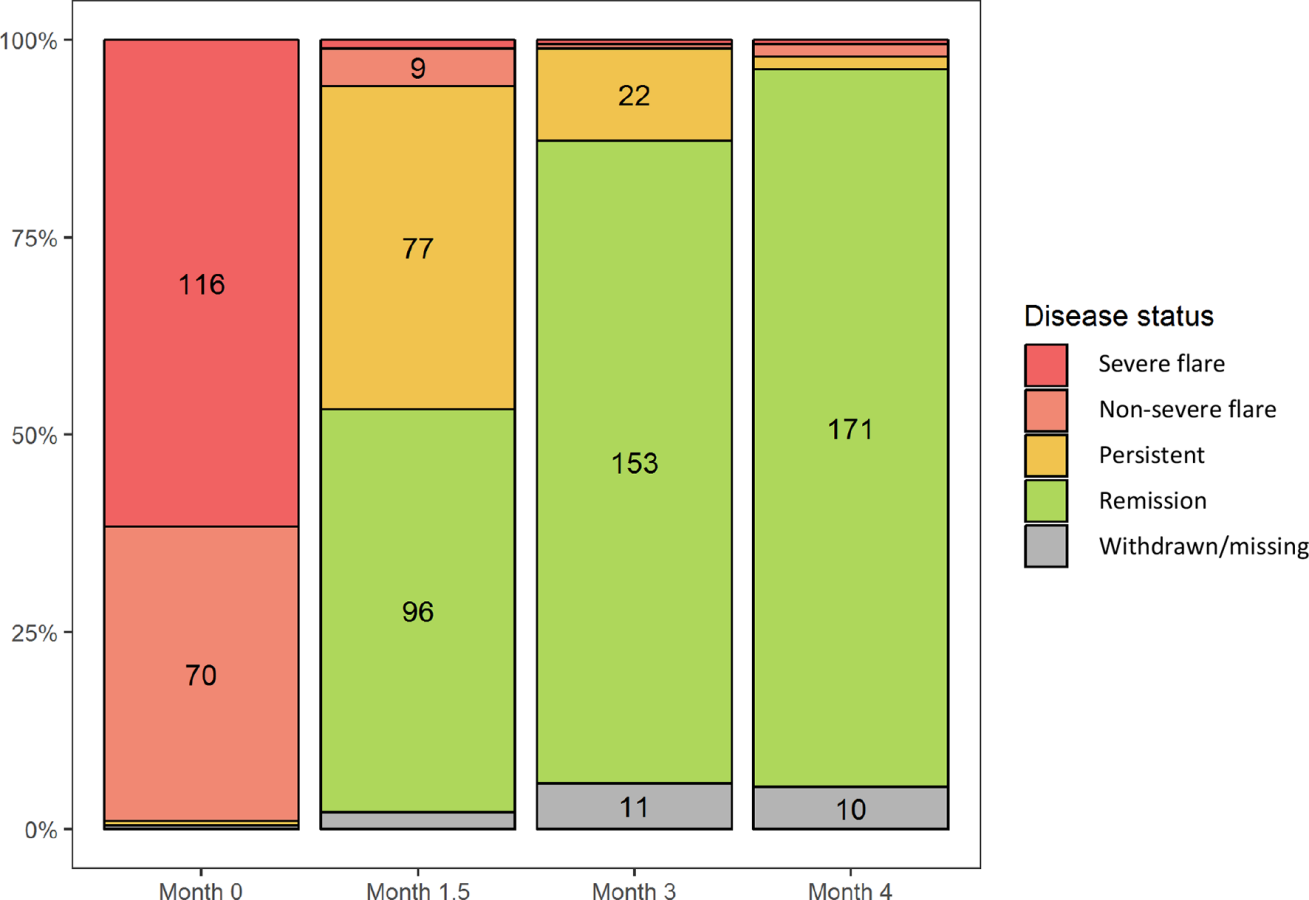
Disease response according to baseline BVAS/WG score. Figures represent the number of individuals according to disease status. In addition to those displayed on the graph: at month 1.5, two individuals had severe disease, and four were withdrawn/missing. At month 3, one individual had severe disease and one limited disease. At month 4, one individual had severe disease, three had limited disease and three had persistent disease. Withdrawn/missing includes all participants who did not attend a study visit either due to death, withdrawal from trial or a missed visit. BVAS/WG, Birmingham Vasculitis Activity Score for Wegener’s granulomatosis.

One hundred and seventy-one out of 188 (90%) patients achieved remission at month 4 (figure 2). Of the 17 patients who did not achieve remission by month 4, 13 (76%) had PR3-ANCA positive disease and 10 (59%) had ear, nose and throat involvement at baseline. Fourteen out of 17 (82%) patients who did not achieve remission had severe (at least one major BVAS/WG item) disease and 5/17 patients (29%) received the higher glucocorticoid dosing regimen. Seven out of 17 (41.2%) non-responders had previously received rituximab, median cumulative dose of 4125 mg (1000–8930), which was comparable with responders (60/171 (35.1%), and cumulative dose 3910 mg (1500–16000)). At month 4, three patients had ongoing ENT disease activity; three had pulmonary manifestations; two had active renal disease; and four had other features of active disease (fatigue,^2^ pachymeningitis^1^ and headache^1^). None of the following baseline variables were predictive of disease response: age, ANCA type at enrolment, glucocorticoid induction regimen, presence of ear, nose and throat or renal involvement (online supplementary table 1), although it is notable non-severe disease was associated with an OR of 2.93, 95% CI 0.915 to 13.1 for subsequent response. Of the 17 patients who did not progress in the trial, only 6/188 (3.2%) had a failure to achieve disease control at month 4, four died in the induction phase, two were withdrawn by their investigator (diagnosis of a new malignancy and occurrence of serious adverse events (SAEs)), three withdrew consent, one required additional therapy not permitted in the protocol and one failed screening and did not receive induction therapy.

10.1136/annrheumdis-2019-216863.supp1 Supplementary data

### Biochemical parameters

**Figure 3:**
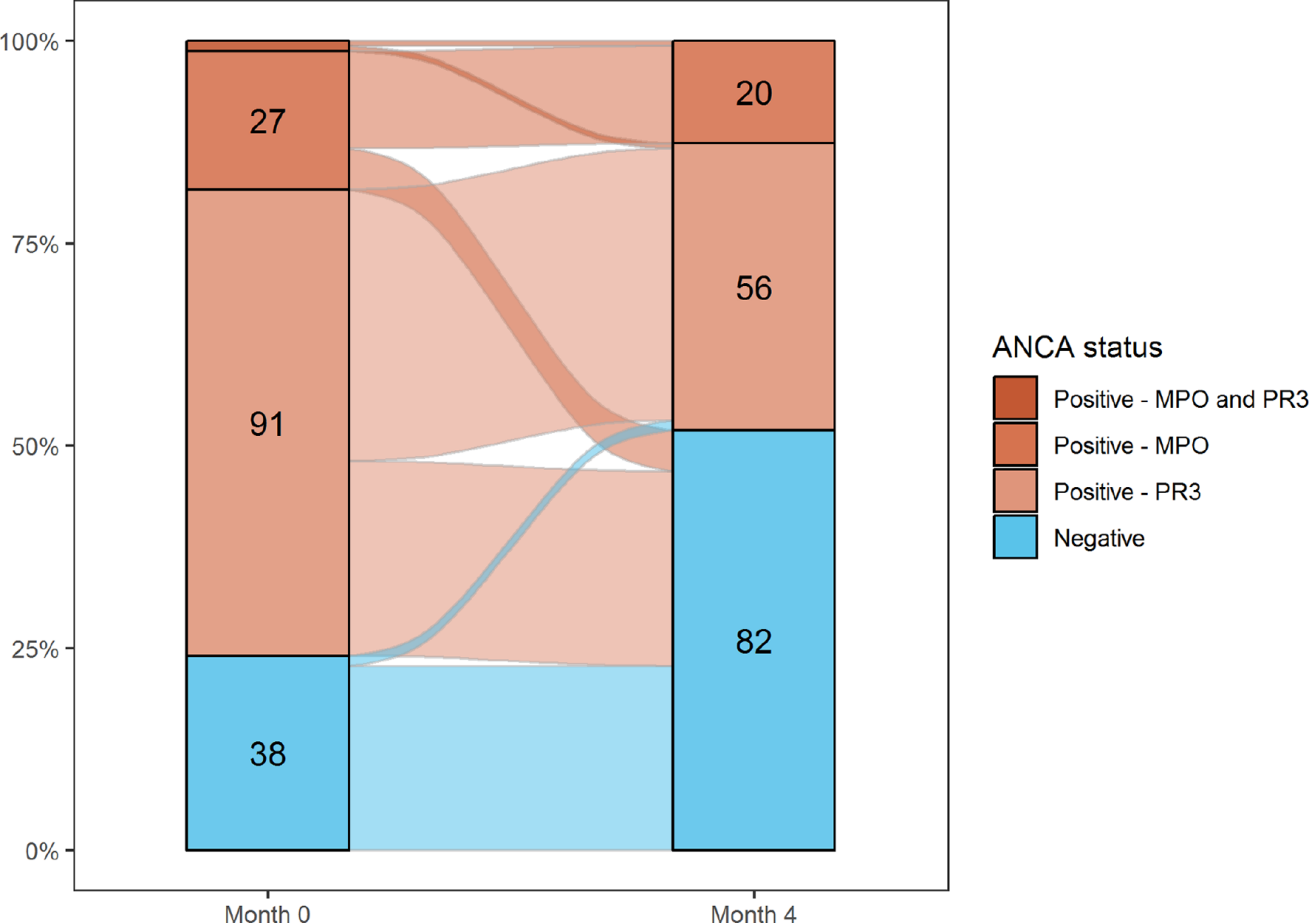
Change in ANCA status between month 0 and month 4 only complete cases reported (n=158). Figures represent the number of individuals according to ANCA status. In addition to those displayed on the graph, two individuals were positive for MPO and PR3-ANCA at month 0. ANCA, antineutrophil cytoplasmic antibody.

Median B cell count fell from 0.12×10^9^/L (12%) (range 0–3.49 (0%–46%)) at baseline to 0×10^9^/L (0%) (range 0–1 (0%–3%)) at month 4. There was no difference in median B cell counts between responders and non-responders. There were modest reductions in C reactive protein levels (median 2.65 mg/L (0–165) at baseline; 1.2 mg/L (0–183) at month 4) and erythrocyte sedimentation rate (21.5 mm/hour (1–149) to 12.5 mm/hour (2–100)) following treatment with glucocorticoids and rituximab. Serum creatinine remained stable (92.5 µmol/L (37.1–472) at baseline and 97.3 µmol/L (42-542) at month 4). One hundred and thirty out of 188 (69.1%) patients tested positive for ANCA at baseline and 81/188 patients (43.1%) at month 4. There was a greater proportion of PR3-ANCA positive patients who became ANCA negative (53.2% to 33.1%) compared with MPO-ANCA patients (14.9% to 12.4%) (figure 3). The two individuals who switched from being ANCA negative at baseline to PR3-ANCA positive at month 4 entered remission.

### Safety

Forty-one SAEs occurred in 27 patients, including 13 severe infections (nine chest, three urinary and one gastrointestinal infection) in seven patients. Five out of 13 infections occurred within 4 weeks of the first induction dose of rituximab. In addition, there were 86 non-severe infections in 59 patients (online supplementary table 2). Fifty-one patients had an IgG level less than 5 g/L at some point during the induction phase (table 2). Four patients (2.1%) died in the induction phase; causes of death included: pneumonia (2), cerebrovascular accident (1) and active vasculitis (1).

10.1136/annrheumdis-2019-216863.supp2 Supplementary data

**Table 2:** Adverse events according to glucocorticoid induction regimen

	Total	1A	1B
Total number (%) of participants with an SAE	27 (14.3)	10 (18.5)	17 (12.7)
Total number (%) of participants with a serious infection	7 (3.7)	0	7 (5.2)
Total number (%) of participants with a non-serious infection	59 (31.4)	12 (22.2)	47 (35.1)
Number (%) of participants with IgG <5 g/L	51 (27.1)	27 (50.0)	24 (25.4)

1A: higher dose glucocorticoid induction regimen, starting at 1 mg/kg/day (maximum starting dose 60 mg/day); 1B: lower dose glucocorticoid induction regimen, starting at 0.5 mg/kg/day (maximum starting dose 30 mg/day).

SAE, serious adverse event.

## Discussion

These data from the induction phase of the RITAZAREM trial, the largest reported prospective cohort of patients with relapsing AAV, demonstrate that rituximab, in conjunction with glucocorticoids, is effective at reinducing remission in patients with AAV who have relapsed, regardless of previous therapy. A high proportion of patients (171/188, 90%) achieved remission by 4 months, and it is notable that 71% of patients received the lower dose glucocorticoid regimen. Although there are retrospective series, the only previous prospective data on induction of remission for this subgroup of patients with ANCA-associated vasculitis was from the RAVE trial that observed a higher rate of remission in 50 relapsing patients treated with rituximab when compared with 50 relapsing patients treated with cyclophosphamide.^7 11–15^ Thus, these data confirm and extend the data on the efficacy of rituximab for relapsing GPA/MPA and supports a recommendation of rituximab for this indication.

The higher remission rate found in RITAZAREM versus RAVE may be due in part to the different definitions of remission. In RITAZAREM, remission was defined as a BVAS/WG ≤1 with a prednisolone dose ≤10 mg/day. The RAVE trial observed a lower remission rate of 64% at 6 months but required a BVAS/WG of zero and complete glucocorticoid withdrawal.^7^ The stricter definition of remission in RAVE, together with differences in trial design, and the enrolment in RAVE of a more severely affected patient population (median BVAS/WG 8.5 (5–13) for patients treated with rituximab), makes direct comparison between RITAZAREM and RAVE difficult. In the current study, only 6 of the 17 patients who did not achieve remission (3.2% of the whole study population) clearly represented failure of the therapy. The remainder were withdrawn from the study protocol either due to investigator or participant decision (seven patients, 3.7%) or died (four patients, 2.1%) within the induction phase. In this cohort, no baseline variables studied were predictive of failure of treatment response, although the small numbers of non-responders make it difficult for such an analysis to be definitive.

Induction regimens in AAV have been associated with high rates of SAEs, and these are more frequent and problematic than failures to control disease activity, thus improvements in the safety of induction regimens are required. In RITAZAREM, SAEs occurred in 14.3% of patients, which is a lower rate than seen in the RITUXVAS trial in which 42% of patients treated with rituximab experienced at least one SAE and the RAVE trial in which 22% of patients experienced at least one grade 3 adverse event.^6 7^

In the treatment of AAV, concomitant use of glucocorticoids is a major contributor to SAEs, especially infective risks, and two glucocorticoid regimens were permitted in this study to suit physician preference. The choice of glucocorticoid regimen was not randomised, and thus may have been subject to bias, so the relative efficacy of these two regimens cannot be completely analysed. Nonetheless, these two regimens appeared similarly effective with the lower dose approach providing approximately two-thirds of the total oral glucocorticoid exposure, and thus reduced dose glucocorticoids can be recommended as a treatment option for this indication.

The key strength of the study lies in the number of patients recruited, making this the largest cohort of patients with relapsing AAV to be studied in a clinical trial, facilitating the collection of high-quality efficacy and safety data on a complex patient population. This is a typical population of patients relapsing with AAV, enriched for patients with PR3-ANCA positivity, with median prior disease duration of 5 years, prior exposure to cyclophosphamide and/or rituximab in the majority and a degree of established chronic damage, meaning that results are broadly applicable. A potential weakness of this study was the option for investigators to choose, rather than randomly assigning the glucocorticoid dosing regimen in a blinded manner. Prescribing practices for use of glucocorticoids in AAV vary, necessitating a pragmatic approach to trial design. However, investigators were required to select the dosing regimen at enrolment, and tapering schedules were standardised.

Achieving a negative serum ANCA test following induction therapy is associated with a lower subsequent risk of relapse in AAV.^16 17^ In the current study, despite 90% of patients achieving remission at month 4, 46% remained positive for serum ANCA at month 4, supporting data from the RAVE trial, in which 53% of patients treated with rituximab remained positive for ANCA at 6 months.^7^ Follow-up in the randomised phase of the RITAZAREM trial will provide further insight into the significance of changes in ANCA levels.

These data from the first phase of RITAZAREM demonstrate that rituximab, in conjunction with even relatively low doses of glucocorticoids, is highly effective at reinducing remission in patients with AAV who have relapsed, with a safety profile similar to or better than previous studies.
