# Supplementary material for: Rituximab as therapy to induce remission after relapse in ANCA-associated vasculitis
Source: Ann Rheum Dis. 2020 Jun 24;79(9):1243–9. doi: 10.1136/annrheumdis-2019-216863 (PMC7456549; doi:10.1136/annrheumdis-2019-216863)
Supplement: Supplementary data [file annrheumdis-2019-216863supp001.pdf]

Supplementary Table 1: Effect of baseline variables on disease response at 4 months (un adjusted regression analysis)

Variable	Estimate	95% CI	P-value
ANCA status at enrolment (anti-MPO vs. anti-PR3)	1.23	0.412-4.54	0.727
Type of relapse (non-severe vs. severe)	2.93	0.915-13.1	0.101
Glucocorticoid induction regimen (1B vs. 1A)	1.04	0.316-2.96	0.948
BVAS/WG score	0.878	0.712-1.1	0.236
Ear, nose and throat involvement (No vs. Yes)	0.924	0.327-2.83	0.884
Renal involvement (No vs. Yes)	1.5	0.534-4.36	0.444
Age (years)	1.02	0.983-1.05	0.339

1A: higher dose glucocorticoid induction regimen, starting at 1 mg/kg/day (maximum starting dose 60 mg/day); 1B: lower dose glucocorticoid induction regimen, starting at 0.5 mg/kg/day (maximum starting dose 30 mg/day).
