# Supplementary material for: Rituximab as therapy to induce remission after relapse in ANCA-associated vasculitis
Source: Ann Rheum Dis. 2020 Jun 24;79(9):1243–9. doi: 10.1136/annrheumdis-2019-216863 (PMC7456549; doi:10.1136/annrheumdis-2019-216863)
Supplement: Supplementary data [file annrheumdis-2019-216863supp002.pdf]

Supplementary Table 2: Line listing of severe adverse events

System Order Class (SOC)	Preferred Term (PT)	Number
**Cardiac disorders**	Acute coronary syndrome	1
	Cardiac arrest	1
**Gastrointestinal disorders**	Abdominal pain	1
	Duodenal ulcer	1
	Gastrointestinal haemorrhage	1
	Intestinal perforation	1
**Immune system disorders**	Vasculitis	3
**Infections and infestations**	Gastroenteritis Escherichia coli	1
	Pneumonia/respiratory tract infection	9
	Urinary tract infection	3
**Injury, poisoning, procedural**	Wound dehiscence	1
**Complications / investigations**	Medical observation	1
**Malignancy**	B-cell lymphoma	1
**Nervous system disorders**	Cerebrovascular accident	1
**Renal and urinary disorders**	Enterovesical fistula	1
	Renal impairment	1
**Respiratory, thoracic and mediastinal disorders**	Laryngeal stenosis	3
**Surgical and medical procedures**	Small intestinal resection	1
**Vascular disorders**	Aortic dissection	1
	Deep vein thrombosis	5
	Pulmonary embolism	3
